# Signalling pathways contributing to learning and memory deficits in the Ts65Dn mouse model of Down syndrome

**DOI:** 10.1042/NS20200011

**Published:** 2021-03-12

**Authors:** Aimée Freeburn, Robert Gordon Keith Munn

**Affiliations:** 1 Discipline of Pharmacology and Therapeutics, National University of Ireland, Galway, Republic of Ireland; 2 Galway Neuroscience Centre, National University of Ireland, Galway, Republic of Ireland

**Keywords:** Down Syndrome, GABA, Hippocampus, Learning and Memory, Ts65Dn

## Abstract

Down syndrome (DS) is a genetic trisomic disorder that produces life-long changes in physiology and cognition. Many of the changes in learning and memory seen in DS are reminiscent of disorders involving the hippocampal/entorhinal circuit. Mouse models of DS typically involve trisomy of murine chromosome 16 is homologous for many of the genes triplicated in human trisomy 21, and provide us with good models of changes in, and potential pharmacotherapy for, human DS. Recent careful dissection of the Ts65Dn mouse model of DS has revealed differences in key signalling pathways from the basal forebrain to the hippocampus and associated rhinal cortices, as well as changes in the microstructure of the hippocampus itself. *In vivo* behavioural and electrophysiological studies have shown that Ts65Dn animals have difficulties in spatial memory that mirror hippocampal deficits, and have changes in hippocampal electrophysiological phenomenology that may explain these differences, and align with expectations generated from *in vitro* exploration of this model. Finally, given the existing data, we will examine the possibility for pharmacotherapy for DS, and outline the work that remains to be done to fully understand this system.

## Cognitive changes in individuals with Down syndrome

On an average, individuals with Down syndrome (DS) on average account for approximately a third of cases of intellectual disability, and typically have lowered IQ scores in general, typically in the range of 30–70 which indicates moderate to mild impairment [1,2]. However, these changes are not uniform: full-scale IQ in individuals with DS can have a large range of scores, despite generally poorer performance on verbal and short-term memory components of these tests [3–8]. DS is often also comorbid with other disorders of executive function or attention such as attention-deficit disorder [6,7]. Adults with DS also often experience disorders of emotion such as depression [8–10], or anxiety disorders such as obsessive-compulsive disorder [11]. Individuals with DS typically have impaired episodic memory [12,13], with impairments in spatial memory of specific interest. Although this is typically assessed as deficits in spatial working memory using an object/location paradigm [14,15], there is evidence that DS is accompanied by poorer long-term spatial memory when allocentric navigation is involved [16]. This constellation of findings implicates impaired hippocampal function as the basis for memory disorder in DS.

## The hippocampal/medial entorhinal cortex circuit and its role in memory and learning

Decades of research have centred the hippocampus and medial entorhinal cortex (MEC) as the neural substrates enabling episodic and spatial memory [17–28]. The discovery of spatially tuned cells in the hippocampus – dubbed ‘place’ cells due to the spatial specificity of their firing [17] – and then of the grid [18], border, direction [19–21], and speed cells [20,21] within MEC led to the idea that these regions function as a cognitive ‘map’ of space. Lesion of the hippocampus tends to lead to more profound spatial memory deficits, while lesion of entorhinal cortex leads to more subtle changes in spatial memory, such as destabilising place cell activity [22]. Inactivation of the hippocampus with GABA agonists does not abolish both places’ cells activity and grid periodicity in the MEC [23], suggesting a strong reciprocal connection between these regions.

## Phasic organisation of information flow in hippocampus and MEC is critical to their function

Local field potential (LFP) in the hippocampus is dominated by the large, regular, 5–12 Hz theta oscillation seen as an animal moves through space. Theta has been demonstrated to be fundamental to normal hippocampal functioning theta in the hippocampus is central to learning and memory. First noted by Winson in 1978; the abolition of theta within the hippocampus is correlated with impairments in spatial navigation [24]. Reduction in hippocampal theta was then rapidly shown to correlate to imapirments in many tasks requiring memory, from spatial alternation [25,26] and reversal [27] to operant tasks [28]. Optogenetic disruption of hippocampal theta while maintaining innervation from the medial septum to the hippocampus produces impairments in spatial performance [29] demonstrating the functional relevance of theta itself to learning and memory.

Ongoing activity within the hippocampus is almost invariably organised relative to theta and is typically phasically entrained to theta rhythm. A core theta phase dependency is the phenomenon of place cell precession: as an animal moves through the firing field of the place cell, it tends to ‘precess’ on the underlying theta wave [30,31], linking spatial information (the location of the animal) to temporal information (the phase of theta). The separate phases of encoding and retrieval (SPEAR) model proposes that spiking of cells at different phases of theta segregates the resultant information flow into encoding and retrieval epochs [32]. Encoding of new associations occurs during the phase of theta during which input from the entorhinal cortex is strongest; while memory retrieval occurs when CA1 input is dominated by CA3 projections [33]. Theta is also a prominent rhythm within the MEC, which also arises from projections from medial septum [34–36]. Grid cells in the MEC have also been shown to precess through theta in each of their firing fields [37–40]. Likewise, reducing theta output from medial septum to MEC results in the regular hexagonal structure of grid cells in MEC degrading. Other cells in the MEC have been shown to be related to theta, with head direction cells being often strongly modulated by theta, and occasionally theta ‘skipping’, such that they are only active on every other theta oscillation [41,42]. Organisation on the theta wave is a clear motif organising the activity of the entire hippocampal/entorhinal circuit. Given the central role of phasic organisation of hippocampal activity in the successful encoding and retrieval information, it is reasonable expect changes in this organisation to be key feature of disorders of learning and memory such as DS. Understanding the flow of information within this circuit, as well as its major inputs, is central to understanding how differences in signalling within the circuit might produce learning and memory deficits.

## Innervation of the hippocampus from medial septum and the origin of theta rhythm

**Figure 1:**
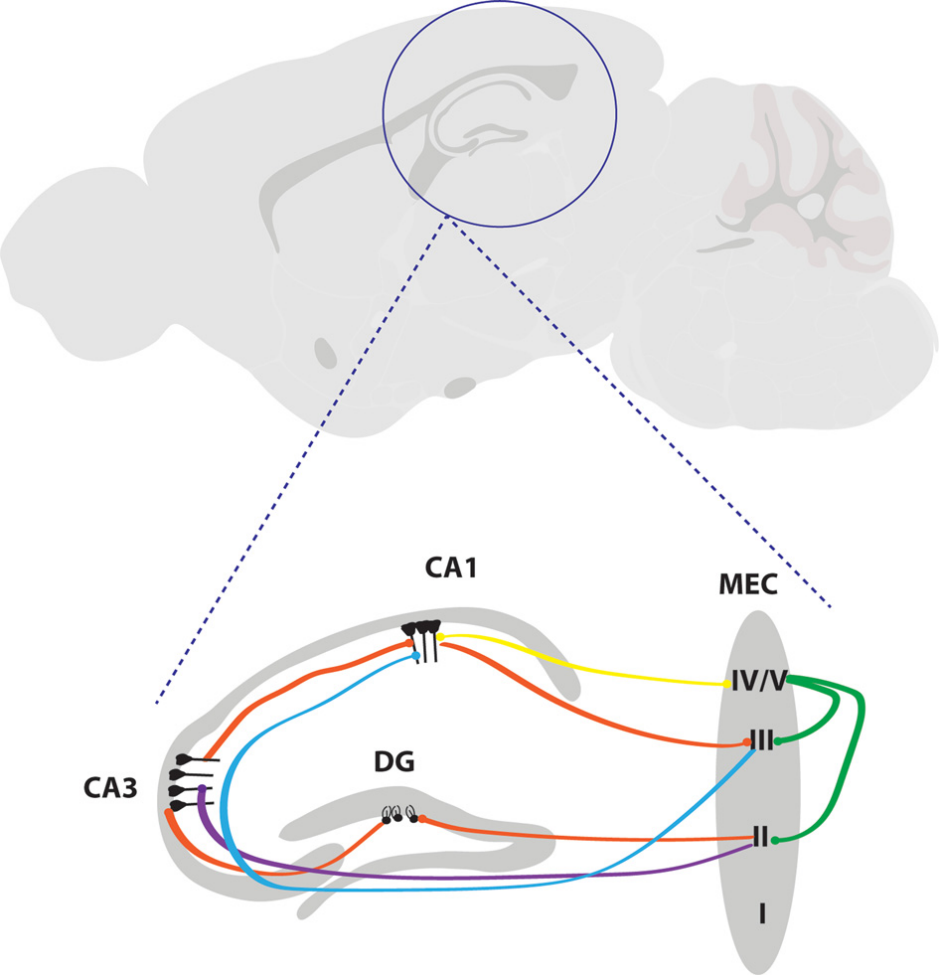
Major connectivity within HPC and between HPC and MEC The most well-known and canonical loop within the hippocampus is the trisynaptic loop (orange) originating from layer II of MEC and travelling via the perforant path to synapse at neurons in the dentate gyrus. From there, the second synapses of the pathway are made at neurons in CA3, and the third at CA1, before the pathway travels back to the MEC layer III via the subiculum. However, another connection to CA3 directly from layer II exists (purple) [67,68], and MEC layer III synapses directly on to neurons in CA1 (the temporoammonic pathway, blue) [69]. Layer IV/V has a reciprocal connection with CA1 (yellow), and intrinsically connects with layers II and III (green) [70].

The hippocampus receives input from a wide range of neocortical inputs. Perhaps the most well-known and well-studied projections to both the hippocampus and entorhinal cortex are the ascending excitatory and inhibitory projections from medial septum. Lesion work has demonstrated that hippocampal theta is conducted via the basal forebrain regions of the medial septum and diagonal band of broca [43–46], and damage to septum not only abolishes theta in the hippocampus, but produces profound spatial memory deficits similar to lesion of the hippocampus itself [24,47,48]. The septum contains cholinergic, GABAergic, and glutamatergic neurons, and all three types send long-range projections to the hippocampus. Several projections to the hippocampus underlie spatial processing (see Figure 1), at the core of which is a ‘trisynaptic loop’ from layer II of the MEC through to the dentate gyrus, CA3, and CA1 in addition to direct, unfiltered projections from MEC layers III/IV to CA1 (Figure 1). A major hippocampal input is comprised of the perforant pathway in which relatively few (∼200000 in rat) EC layer II cells diverge into relatively many ∼1000000 granule cells in the dentate gyrus, enabling the ‘pattern separation’ of memory into encoding cues such as ‘place’, ‘size’, and ‘colour’. Mossy fibres from the dentate gyrus then converge into ∼160000 pyramidal cells in the CA3 field, which are highly reciprocally connected. CA3 is where auto-associative ‘pattern completion’ occurs to translate representative cues into unique, stable episodic memories which can be easily recalled following future cues [49–52]. This information is fed forward to area CA1, in which stable place representations are formed, modified, and then fed out of the hippocampus.

A major relay of hippocampal theta is the medial septal nucleus, which sends dense excitatory cholinergic [53] and glutamatergic [54,55] connections to the hippocampus and parahippocampal regions. It also sends inhibitory GABAergic projections to these regions [56]. While all three projections innervate the hippocampus, there are some fundamental differences in their patterns of connectivity: GABAergic projections from MS tend to target inhibitory interneurons in the hippocampus, particularly in area CA1 [57]. The phasic firing of interneurons within the hippocampus is thought to synchronise the firing of pyramidal cells [58]. Learning and spatial memory appear to critically depend on these GABAergic projections [59,60], while both cholinergic and GABAergic projections from the medial septum are essential for hippocampal theta rhythm [61]. However, the origins of theta rhythm do not lie in the medial septum, but rather information from various other brain nuclei coding for frequency and amplitude which are integrated in medial septum and relayed on. Careful dissection of this circuit has demonstrated that the frequency of hippocampal theta rhythm is dependent on the posterior hypothalamus, which is through to control the gain or amplitude of theta [62]. Input to the medial septum from the supramammiliary bodies [63,64] and the reticular formation [65,66] have also been implicated as important inputs in theta generation.

## Animal models of DS

**Figure 2:**
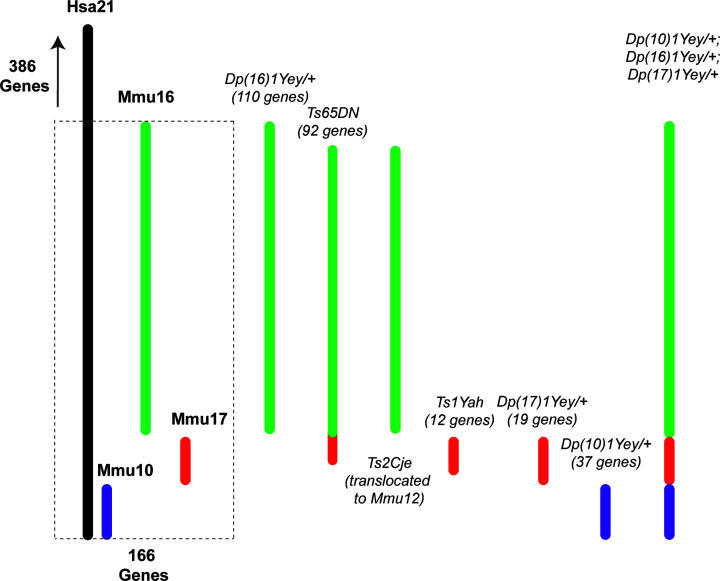
Comparison of the genes involved in mouse models of DS Hsa21 comprises 552 genes in its long arm, and 166 of these (dashed line) are homologues to chromosomes found on murine (Mmu) chromosomes 10 (purple),16 (green), and 17 (red). Unlike other models of DS, the TS model recapitulates almost completely the anatomical, neurobiological, and behavioural phenotypes of human DS including deficits in cognition and memory, neuroanatomical characteristics, low life expectancy and age-related cognitive decline reminiscent of Alzheimer’s disease (AD) [75–77]. However, a drawback of the TS mouse is that it carries three extra copies of an extra segment with non-DS related genes arising from Mmu17, including ∼35 protein-coding genes. Various other mouse models have been developed that involve trisomy of chromosomes 10, 16, and 17 [78–81]. The Ts2Cje mouse involves the same portion of Mmu16 as is triplicated in the Ts65DN mouse, but this sequence is translocated to Mmu12. The TS mouse has relatively good construct validity, triplicating a large number of the same genes as seen in human DS, but its construct validity is not perfect due to incomplete triplication of all triplicated Hsa21 genes and its inclusion of non-triplicated genes in DS on the trisomic portion of Mmu17. This being the case, it has long been considered the best DS model available due to its extremely high, face validity, as both the structural and behavioural differences seen in this model map most closely to human DS (Tables 2 and 3). A more recently developed model combining the Dp(10)1Yey/+, Dp(16)1Yey/+, and Dp(17)1Yey/+ models has produced a promising alternative model to Ts65Dn, possessing very high construct validity, and the same learning and memory deficits seen in DS and the TS model [82].

The pathogenesis of DS is poorly defined. DS is a contiguous gene syndrome that spans 35 Mb of the long arm of human chromosome 21 (Hsa21), resulting in increased expression of a subset of encoded genes. An extra copy of one or more genes or regulatory sequences on Hsa21 and associated underlie the DS phenotype [71]. A multiplicity of transgenic mouse models for candidate genes has been developed to study of the complex genotype–phenotype interactions, identification of dosage-sensitive genes and potential therapeutic targets (Figure 2). However, orthologues of Hsa21 map to differing segments across mouse chromosomes – primarily Mmu16, followed by internal segments of Mmu17 and Mmu10, which makes trisomy 21 difficult to model (Table 1). Illuminates the extant mouse models for DS and murine chromosome segments that are triplicated in each. While Hsa21 encodes for 161 protein-coding genes and 5 microRNAs, only 157 of these are conserved in the mouse [72]. Likewise, some murine genes do not have human homologues (i.e. Itg21 located within the Hsa21 homologous region) which may carry results in genetic modifications which may undermine accurate DS phenotyping [73].

**Table 1:** Mouse models that involve triplication of segments of specific murine chromosomes

Triplicated chromosomes(s)	Mmu 16 segment	Mmu 17 segment	Mmu 10 segment	Mmu 16,17, and 10 segments
Mouse models	Dp(16)1Yey/+ Ts65DN	Ts2Cje, Ts1Cje Ms1Ts65 Ts1Rhr Ts1Rhr Dep(17)1Yey/+ Ts1Yah	Dp(10)1Yey/+	Dp(10)1Yey/+; Dp(16)1Yey/+; Dp(17)1Yey/+

**Table 2:** Comparison of neuronal changes observed in human DS and the Ts65DN mouse model

	DS (Human)	Ts65DN
**Brain volume**	↓	↓
**Neuronal density**	↓	↓
**Cerebellar volume**	↓	↓
**Neurogenesis**	Slow cell cycle	Slow cell cycle
	Impaired proliferation of neural precursors	Impaired proliferation of neural precursors
	Impaired differentiation	Impaired differentiation
**Dendrite morphology**	Reduced density	Reduced density
	Abnormal morphology	Abnormal morphology
**Electrophysiology**	EEG abnormalities EEE coherence abnormalities	EEG abnormalities EEG coherence abnormalities impaired long-term potentiation

Adapted from [83].

**Table 3:** Summary of the behavioural similarities between human DS and mouse models of DS

	DS (Human)	Ts65Dn	Dep(17)1Yey/+	Dep(10)1Yey/+	Ts1Yah
**Spatial context discrimination**	↓	↓	=	=	
**Spatial learning and memory**	↓	↓	=	=	↑
**Working memory**	↓	↓			

Ts65Dn animals share many similar deficits (down arrow) in learning and memory with human DS compared with control, while some other mouse models are either unaffected (=) or enhanced (up arrow) compared with control. Adapted from [78].

The TS65Dn model was the first viable segmental trisomy model for DS [84], and involves trisomy of the distal portion of chromosome 16 in the TS model results in extra copies of ∼115 genes homologous to Hsa21. Among replicated genes is the amyloid precursor protein (APP) gene – a primary genetic substrate of early-onset Alzheimer’s disease (AD) and BFCN loss in DS patients [71,74].

Nevertheless, the Ts65Dn model remains the leading mouse model of DS, owing to its relatively uncomplicated genetic triplication, its wide availability, and its very high face and construct validity. In the following sections, as we consider changes in the Ts65Dn mouse in the context of human DS, it will become apparent that the central features of DS are well replicated in the Ts65Dn mouse, confirming its place as the eminent mouse model of DS.

## Behavioural differences in the Ts65Dn model

In order to be a good model for human DS, a mouse model should not only triplicate candidate genes for the disorder, but should recapitulate the behavioural phenotype of human DS. One of the most striking differences in human DS, and perhaps one of the most problematic to those with DS, are difficulties with learning and memory [16,84–87]. Crucially, allocentric spatial memory, perhaps the primary measure used to study learning and memory in rodents, is impaired in individuals with DS, allowing us to easily probe the Ts65Dn model for similarities in behavioural output demonstrating impaired memory.

### Learning and memory

Since the inception of the Ts65Dn model, it has been observed that the performance of TS animals in classic tests of spatial memory such as the Morris Water Maze is impaired [88–92]. In this test, depending on the exact protocol, animals must learn the position of a hidden platform in a water-filled maze, and then must remember the location of the platform on subsequent training days. Ts65Dn animals show impaired learning – they take longer to find the platform throughout training compared with control. A probe test with the platform absent tests recollection of the location of the platform. Ts65Dn animals spent less time searching the correct quadrant of the maze compared with control. Further investigation has demonstrated that Ts65Dn animals are impaired in a wide array of behavioural tests. They tend to perform poorly relative to non-trisomic animals in tests of context discrimination [93] in which animals remember a spatial location in which they previously received a footshock, as well as in tests of working memory [94] and novel object recognition [95,96]. Olfactory learning in Ts65Dn mice is also impaired [97]. Ts65Dn learned the radial arm maze significantly more slowly than control animals, and performed worse in a test of their delayed retention of this task [98], in a dry-land recapitulation of their learning and memory deficits uncovered in the Morris Water Maze. This constellation of behavioural deficits are classically reminiscent of those caused by hippocampal damage in both rodents and humans, and implicate the hippocampal-entorhinal region in the signalling abnormalities central to this model in specific, and DS in general. There is one important caveat to consider in the interpretation of memory deficits in the Ts65Dn model. In order for differences in behavioural tests of learning and memory to be correctly interpreted, other explanations for differences in behaviour between DS model animals and control animals must be ruled out. One of the most obvious potential causes of differences between groups in tests like the Morris Water Maze are differences in the motor output of the animals. Indeed, differences in nociception and proprioception also appear to be a central change in the Ts65Dn model. Ts65Dn animals show decreased responsiveness to behavioural tests of pain [99], and show a variety of subtle deficits in fine motor control, including abnormal paw-print placement that indicated less regularity in stride combined with shorter stride length [100,101]. Ts65Dn animals also spend less time balanced on the roto-rod, and slower and more variable swimming speed in the water maze [100]. Some of these differences in motor control may be due to altered muscle development in Ts65Dn [102,103], but this model also shows altered spinal nerve development [104]. It is also possible that these changes arise from divergence in the central generation of motor output and reception of the motor efferent copy. Whatever the cause, these behavioural differences must be considered when interpreting differences in task performance between Wildtype and Ts65Dn animals. In spite of these caveats, the array of different tests demonstrating clear impairments in memory in the Ts65Dn animal (some unlikely to be contaminated by the aforementioned differences) make it very unlikely that these changes in motor output can account for all of the deficits seen in Ts65Dn animals.

## Neuronal pathways altered in the Ts65Dn model

The observation that individuals with DS develop Alzheimer’s-like pathology at rates far greater than the general population prompted much interest in putative differences in the neurotransmitter systems known to be involved in this pathology between DS and unaffected individuals. Cholinergic dysfunction is one of the central changes in AD. Individuals diagnosed with AD show a loss of cholinergic neurons in the basal forebrain [105–107]. In a similar manner to humans with AD-like pathology, the Ts65Dn mouse shows marked reduction in the number and size of cholinergic neurons in the basal forebrain [108–110]. thought to be the result of impaired retrograde transport of nerve growth factor (NGF) [111] As might be expected, the number of cholinergic neurons correlates with scores of attention [109]. Interestingly in this model, the number of cholinergic neurons in the basal forebrain decreased in with age in control animals, but the number of neurons did not decline further in the Ts65Dn animals as they aged, suggesting that the attentional deficits that appear with AD-like pathology are related directly to changes in basal cholinergic neurotransmission. This deficit in cholinergic transmission in the Ts65Dn model appears to be neurodevelopmental. Intraventricular delivery of NGF reverses the abnormal neuronal number of cholinergic neurons in the basal forebrain [111], and choline supplementation *in utero* appears to both normalise the number of cholinergic neurons in the basal forebrain in the resultant offspring in adulthood, and to some extent might rescue the learning and memory deficits associated with impaired cholinergic transmission [112]. Choline supplementation has been shown to affect the RNA expression in CA1 of key genes involved in calcium signalling and synaptic plasticity, indicating a long-term rescue by this intervention [113]. Recent evidence has emerged that suggests the picture of impaired cognition associated with reduced cholinergic signalling from the basal forebrain is more complicated. Pharmacological treatment with the acetylcholinesterase inhibitor Donepezil increases the level of acetylcholine, but does not affect learning and memory in the Ts65Dn model. By contrast, the GABA(A) antagonist Pentylenetrazole produced a robust increase in learning and memory performance by these animals [77]. Since the major outputs from the medial septum to learning and memory critical regions such as hippocampus and entorhinal cortex are a push-pull mix of excitatory cholinergic and inhibitory GABAergic projections (with a relatively recently uncovered sizable minority glutamatergic projection [55]), it seems likely that the ratio of cholinergic to GABAergic output is critical to maintaining normal activity within hippocampus. Indeed, cholinergic activation has been postulated to control the ‘gain’ or power of hippocampal theta rhythm through tonic activation of hippocampus, while GABAergic activity may control the frequency through inhibitory or disinhibitory action on hippocampal interneurons [114]. In addition to reduced cholinergic output from the basal forebrain in Ts65Dn animals, there is a marked up-regulation in choline transferase (CHAT) in regions receiving output from this region such as hippocampus and temporal cortex, suggesting a compensatory mechanism to reduced basal acetylcholine input [115].

Given the central involvement of output from medial septum in the generation of theta rhythm and the central alterations of key neurotransmission systems in septum in DS models, it is reasonable to expect changes in key aspects of hippocampal rhythmicity in DS models. This expectation is emerging as the case. Very recently theta power was shown to be increased in Ts65Dn mice during sleep [116]. The frequency of hippocampal theta and the phasic relationship of hippocampal theta to gamma oscillations has very recently been shown to be altered in the Ts65Dn model [117]. Moreover, the firing of CA1 place cells in the Ts65Dn model has been shown to be abnormally phase-locked to the trough of theta, in contrast with control cells, which precess normally through all phases of theta [117]. These changes may explain the mechanism of the learning and memory deficits in this model. The routing of information into encoding and retrieval epochs is thought to be tightly controlled by the phase of theta at which spiking occurs [32], and long-range communication between hippocampus and entorhinal regions is thought to depend on gamma oscillations, which are likewise phasically entrained to theta [118–121].

### Changes in inhibitory GABAergic connections

**Figure 3:**
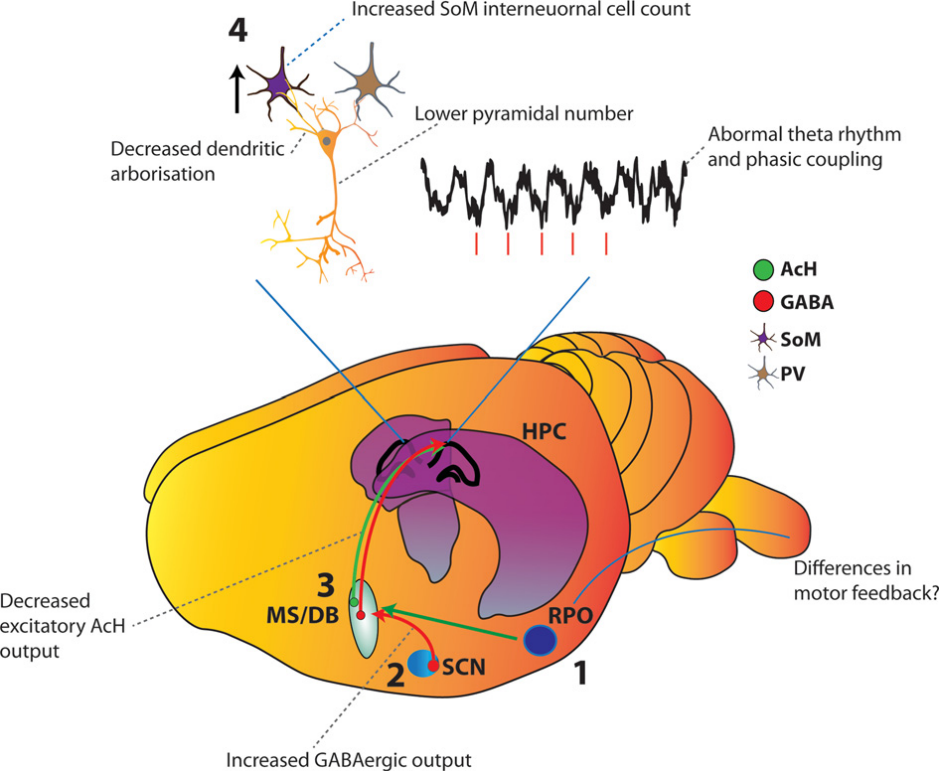
Summary of theta generation mechanisms and known signalling changes in the Ts65Dn mouse (grey text) The motor efferent copy arrives in the reticular formation (1) where it is relayed by excitatory projections from the medial supramammiliary nucleus and posterior hypothalamus to the medial septum [62,64,136–138]. The resulting theta rhythm is relayed to the hippocampal formation by GABAergic and cholinergic output from septum (3). In DS, GABAergic output from the suprachiasmatic nucleus to the medial septum is enhanced. (2) Cholinergic output from the basal forebrain (inc. medial septum) is diminished, and this tends to increase with age. This alters the excitatory (green)/inhibitory (red) output balance of the medial septum, resulting in excess inhibitory control. This is coupled (4) with increased connectivity of somatostatin-positive interneurons with hippocampal neurons and reduced dendritic arbourisation and cell loss among pyramidal neurons, which leads to abnormal theta rhythm within the hippocampus, and increased phase-locking of place cells to the ongoing theta wave.

A very recent finding shows that the phenomenology of interneurons in Ts65Dn mice are also changed; the inhibitory connections between somatostatin positive Martinotti cells and excitatory pyramidal cells are dramatically strengthened in the Ts65Dn animal, and parvalbumin positive interneurons become more excitable, but lose their tendency to enter fast-spiking epochs [122]. Critically, these changes were seen to increase the tendency of pyramidal cells to become phase-locked to network oscillations. Other work has shown an overall increase in inhibitory interneurons within the hippocampus; the changes again selectively sparing parvalbumin-positive interneurons [123]. Indeed, genetic down-regulation of GABA(A) receptors expressing the α-5 subunit, which are primarily found in the hippocampus [124,125]. improves both the structure and phenomenology of the hippocampus in Ts65Dn animals, it rectifies some of the behavioural differences in these animals [126]. Figure 3 summarises these changes in signalling.

### *In vitro* signalling

Plasticity within the hippocampus in the form of long-term potentiation (LTP) and long-term depression (LTD) have (not uncontroversially) been mooted as neural substrates of learning and memory [127]. In the Ts65Dn mouse, LTP in hippocampus is dramatically reduced [128–130], while LTD is facilitated [131]. Once again, an excess of inhibition, or at least a change in the excitation/inhibition balance is central to these changes [132,133], inhibitory post-synaptic potentials mediated by both GABA(A) and GABA(B) are more efficient in the Ts65Dn model [134]. Inverse agonism of GABA receptors expressing the α-5 subunit reverses these changes [135]. Another study, while failing to find differences in LTP between trisomic and disomic animals using high-frequency stimulation found a profound reduction in LTP induced by theta-burst stimulation in Ts65Dn animals [129]. Theta-burst LTP induction is sensitive to picrotoxin, underlining the role of altered GABA signalling in the hippocampus in the changes in hippocampal phenomenology seen in the Ts65Dn mouse.

Based on the dual roles of altered cholinergic input to the hippocampus and GABA signalling within the hippocampus, both systems appear to be mutually synergistic in producing the cognitive and behavioural deficits in the Ts65Dn model. It seems likely that reduced cholinergic output from the basal forebrain alters the finely tuned excitatory/inhibitory balance upon which septal-generated theta depends, and this change alters the frequency of the oscillation being generated by septum. This frequency disturbance, in combination with changes of the activity of interneurons in the hippocampus then likely cause all aspects of hippocampal function that depend on theta to organise their information flow to now fire at the wrong phase, and hence route their information suboptimally. This reduction in computational efficiency then likely causes the impairments in learning and memory central to DS.

## Structural changes in the brain of Ts65Dn mice

One of the earliest discovered neural correlates of intellectual disabilities in humans is a pronounced change in the dendritic arbourisation of excitatory pyramidal cells in the cortex; [139] their dendritic arbour tends to be diffusely abnormal, either abnormally long or short, or a marked reduction in number. These differences in connectivity between neurons suggests a difference in neuronal contact is a key difference in DS. Further studies imply that these differences in dendritic morphology represent a developmental disorder; normal neuronal development goes awry during postnatal development. There is either increased branching of, or no difference in [140] the dendrites of pyramidal cells in layer III of prefrontal cortex of 2.5-month-old infants, but infants older than 4 months show marked shortening of the dendrites in this area [141]. Interestingly, ablation of the excitatory input from entorhinal cortex to the hippocampus causes dendritic atrophy in the granule cells in dentate gyrus reminiscent of the overall changes in DS, [142] suggesting that the origin of the neuronal dendritic changes in DS is a damping of excitatory input. Indeed, the dendritic spine protein drebrin is reduced in human DS, and in another similarity with AD, in the brains of patients with Alzheimer’s [143]. One of the central hypotheses concerning these changes is that there are differences in cellular development in DS from very early in life that produce abnormalities in cell-to-cell connectivity through contact at dendritic spines. While this may or may not result in structural abnormalities in spine formation and appearance, the underlying differences in cell-to-cell signalling nevertheless eventually causes connective failure of the synapses between neurons and a negative feedback cycle producing retraction of the dentritic contact. In the case of DS, the reduced excitatory contact between cells due to reduced cholinergic signalling may be the root cause of this connective failure. This constellation of differences could account for the key changes in cell number, arbourisation, and cell-to-cell contact seen in the Ts65DN mouse, and may be the key neurodevelopmental change in human DS.

### Differences in hippocampal signalling

While it is clear that DS in mice is associated with a marked loss of neurons in the basal forebrain, and specifically in the medial septal area, there are also changes in other regions. While organisation of activity in hippocampus and between hippocampus and associated regions is abnormal in the Ts65Dn model, the physical structure of the hippocampus is likewise altered in this model. While the overall hippocampal volume of Ts65Dn mice is largely similar to control, Ts65Dn mice have many fewer granule cells in the dentate gyrus of the hippocampus and more neurons in region CA3 [144]. Other brain regions show similar changes. Olfactory piriform cortex in Ts65Dn animals contains neurons that are not only less dendritically arbourised; they make more inhibitory and fewer excitatory connections [145]. In temporal cortex, the selective reduction in number of asymmetric synapses and the increased synaptic zone of contact in Ts65Dn animals suggests a specific reduction in excitatory connections, and an attempt by the system to compensate by increasing the contact area of each synapse [146]. More recently, reductions in neuron density have been seen throughout the hippocampus in the Ts65Dn model, with lower neuronal number in CA1, and reduced neuron-to-synapse ratio in regions CA1, CA3, and dentate gyrus. In a strikingly similar manner to the rest of temporal cortex, there is a selective reduction in asymmetric synapses, suggesting again a selective reduction in excitatory contact [147]. While it is possible that these changes result from the primary changes, which appear to be reductions in cholinergic input from the basal forebrain, changes in the dentate gyrus, at least, seem to be due to failed cell proliferation and survival during development [148]. Adrenergic agonism has been shown to be effective in supporting the proliferation of granule cells in the dentate [149–151]. Indeed, very recent data have shown that neonatally treating Ts65Dn mice with a β-2 adrenergic agonist successfully restores normal dendritic development in hippocampal granule cells [150], suggesting that a prolonged lack of normal connectivity produces the abnormal dendritic arbourisation. Interestingly, this prophylactic treatment does not rectify the abnormal neurogenesis in the Ts65Dn model, although β-2 agonism was shown to increase neural proliferation *in vitro*. This difference suggests a multifactorial etiology for abnormal neural development in DS, and a putative degenerative cycle – a reduction in excitatory input causes a reduction in dendritic arbourisation, which in turn further alters the excitatory/inhibitory balance.

### Origins of abnormal excitatory input to the hippocampal formation

Novel object recognition memory is impaired in Ts65Dn mice, but is restored by lesion of the suprachiasmatic nucleus [152]. This surprising finding aligns with behavioural data in humans with DS that show dysfunctional sleep is a central aspect of the syndrome [153–156]. Surgical ablation of the suprachiasmatic nucleus of Siberian hamsters produces DS-like learning and memory deficits in object recognition memory [157]. Critically, treatment with a GABA(A) antagonist restores object recognition memory to SCN-ablated hamsters [157]. Cholinergic neurons in the basal forebrain project to the SCN [158–160], and loss of this cholinergic input in DS and AD is likely to underlie the sleep problems common to both disorders. Paradoxically, lesion of the SCN in Ts65Dn animals restores their object recognition memory [152]. Major, but somewhat overlooked, outputs of the SCN are the septal nuclei [161]. The output of the SCN is largely GABAergic. One of the major hypotheses that has emerged is that the origin of the cognitive changes in Ts65Dn animals is increased GABAergic input to MS from SCN, and in turn reduced cholinergic output to HPC and EC from MS [157,162–164]. This reduction in cholinergic output may then upset the excitatory/inhibitory balance critical to theta regulation, altering the frequency of theta, upsetting the phasic relationship of hippocampal information flow, and reducing the efficiency of the learning and memory system. Within the hippocampal formation specifically, this damping of cholinergic input causes a reduction in the dendritic arbourisation of granule cells in the dentate gyrus, which reduces the excitatory signal still further. The reduced excitatory input travelling along the trisynaptic loop to CA1 meets a normal amount of GABAergic inhibitory input from local interneurons, and the signal is damped still further. It is possible that these changes lead to plastic changes including the loss of neurons within the hippocampus, and that these changes lead to the difficulties in learning and memory seen in this disorder. If the central difference is an alteration of the excitatory/inhibitory balance, the question of whether pharmacological rectification of this imbalance can reverse these changes is of key importance.

## The potential of pharmacotherapy for DS and future directions

The balance of evidence is that the deficits in learning and memory seen in DS result from an alteration of the excitatory/inhibitory output balance from medial septum to hippocampus and associated rhinal cortex regions, coupled with changes in the interneuron architecture within the hippocampus [130,132]. This observation suggests that pharmacotherapy addressing this altered balance may have therapeutic potential. Animal models have so far shown that a single bolus of GABA(A) antagonists such as picrotoxin and pentylenetetrazol can reverse deficits in learning and memory in the Ts65Dn mouse [77,95], and that these effects can last for a much longer time than the drug persistence, suggesting an intermediate-term neuroplastic change. Clinical use of GABA antagonists in humans remains problematic due to the lowering of the seizure threshold and increased risk of seizure that results from GABA antagonism. Given the central changes in DS seem to result from globally increased GABAergic signalling and a reduction in the excitation/inhibition ratio, however, this caveat may be less clinically relevant in a DS population. Newer drugs that are selective for α-5 subunit containing GABA receptors offer some clinical benefit, as this subclass of GABA receptors has been shown to be highly involved in the cognitive deficits in the Ts65Dn model [126]. It is possible that these agents could produce the pro-cognitive effects of GABA antagonism with less of the seizure risk [165] While antagonism of GABA(A) receptors shows clear pro-cognitive promise in DS, the Ts65Dn mouse model also shows enhanced GABA(B) transmission in both the dentate gyrus and area CA1 of the hippocampus [130,134,166], offering a potential novel mechanism to treat learning and memory deficits in DS without the seizure liability. Another potential treatment is the indirect modulation of GABA neurotransmission through modulation of the 5-HT system. Chronic serotonin-specific reuptake inhibitor (SSRI) treatment modulates GABA transmission [167], and in the Ts65Dn mouse model, treatment early in life with the SSRI fluoxetine can prevent abnormal development of the hippocampus, and normalises hippocampus-dependent memory [168]. However, other evidence demonstrates a lack of pro-cognitive effect of adult SSRI treatment in the Ts65Dn model, and an increase in seizures and mortality [169], suggesting that early-life treatment may prevent neurodevelopmental changes in the Ts65Dn model that, after they have occurred, are resistant to subtle or indirect changes in GABA signalling. Given the modulation of 5-HT by GABA [170] in a synergistic feedback loop, altered GABA neurotransmission in the Ts65Dn model early in life may also change the 5-HT system.

The balance of evidence regarding the signalling changes in DS that result in learning and memory deficits seem to be able to be summarised as a change in the excitatory/inhibitory balance that results in abnormal function of the hippocampus. These chronic changes in excitatory balance produce long-term developmental changes that result in a variety of morphological changes in the hippocampus; an increase in interneuronal contact, a reduction in the dendritic arbourisation of pyramidal neurons, and an overall reduction in cell number. There is preliminary behavioural evidence that correcting the excitatory/inhibitory balance corrects to some degree the learning and memory aspects of cognitive function in DS. What is currently unclear is whether this pharmacological therapy restores normal hippocampal function, or whether this treatment corrects the many neurodevelopmental differences we see in DS. Future experiments could clarify these questions, and could clarify to what extent the changes in DS are reversible, and whether these changes are all critical for the learning and memory deficits in DS to be seen. Future research could also clarify if it is only pharmacological damping of the GABA signal that is effective, or if other mechanisms of increasing excitation may also be therapeutically relevant, opening myriad new potential pharmacotherapies for DS.

## Abbreviations

5-HT: Serotonin
AD: Alzheimer’s disease
DS: Down syndrome
GABA: Gamma aminobutyric acid
HPC: Hippocampus
Hsa21: human chromosome 21
LTD: long-term depression
LTP: long-term potentiation
MEC: medial entorhinal cortex
NGF: nerve growth factor
SCN: Suprachiasmatic nucleus
SSRI: serotonin-specific reuptake inhibitor
